# 76 Osteogenesis imperfecta: a case report on 4 cases

**DOI:** 10.1093/rheumatology/keac496.072

**Published:** 2022-10-07

**Authors:** A Ferhat, F Benaziez, A Rahmoune, N Bahaz, C Dahou-Makhloufi

**Affiliations:** Rheumatology Department of the Bab-El-Oued University Hospital, Algiers, Algeria

## Abstract

**Background:**

Osteogenesis imperfecta, or “glass bone disease”, is a rare genetic disorder due to an abnormality in the production of type 1 collagen. It is characterised by bone fragility and low bone mass, leading to repeated fractures. Osteogenesis imperfecta (OI) can be classified into several types ranging from lethal at birth to benign forms that may be discovered incidentally in adulthood.

We report four observations of OI that caught our attention.

Observation 1: Mr H.A, aged 15 years, with no particular pathological history (atcd), he presented since the age of 4 years old iterative low-energy fractures (06 fractures). The clinical examination finds dorsal scoliosis, deformities of the long bones, blue staining of the sclerae. Radiography showed diffuse bone hypertransparency, thinned cortices and curvature of the long bones. Osteoporosis with a Z-score of -3.8 DS in the spine and -3.1 DS in the femur on bone densitometry (BMD). The patient received 20 courses of pamidronic acid at a dose of 07 mg/kg/year with good clinical (decrease in the number of fractures) and osteodensitometric improvement and good tolerance.

Observation 2: Mr D.A, 16 years old, without any particular pathological atcd, the onset of the disorders goes back to the age of 13 years marked by the installation of repeated fractures following minimal trauma. The patient presents with growth retardation, deformities of the long bones and blue sclera. Radiography showed diffuse bone hypertransparency, thinned cortices, curvature of the long bones with bilateral coxa profonda. BMD showed osteoporosis with a *Z* score of -4.8DS. He was treated with pamidronic acid at 07 mg/kg/year (09 courses) with satisfactory improvement and good tolerance.

Observation 3: Mr A.N aged 41 years with no particular pathological atcd. The history of the disease shows repeated low-energy fractures (06 fractures) since the age of 11 years. On clinical examination, there is an exaggerated kyphosis, blue sclera and bilateral hearing loss on the right. On imaging, diffuse bone hypertransparency, thinned cortices, curvature of the long bones and right acetabular protrusion were noted. On BMD, osteoporosis with a Z-score of -4.0 DS at the spine and femur. The patient received zoledronic acid 04 mg/06 months. The patient improved well with a decrease in the number of fractures and an improvement in bone densitometry.

Observation 4: Mrs B.Z aged 40 years without any particular pathological atcd, having made iterative low energy fractures since the age of 01 month (05 fractures). The patient presents a dorsal scoliosis, deformations of the long bones, growth retardation, blue sclera and translucent teeth. The radiograph shows diffuse bone hypertransparency, thinned cortices and curvature of the long bones. BMD and adequate treatment were not done as the patient was lost to follow-up.

**Conclusion:**

Osteogenesis imperfecta is a rare disease with a polymorphous clinical expression. Late diagnosis delays the initiation of adequate treatment and consequently the persistence of fractures. Bisphosphates improve bone mass and reduce the incidence of fractures and are generally well tolerated.

